# K-means clustering applied to vegetation indices for mapping cultivated areas using high-resolution Moroccan Mohammed VI satellite imagery

**DOI:** 10.1038/s41598-026-41167-1

**Published:** 2026-02-25

**Authors:** Abdellatif Moussaid, Mohamed Bayad, Yousra Gamoussi, Yassir El Hamdouni, Hamza Briak

**Affiliations:** 1 Center for Sustainable Soil Sciences (C3S), College of Agriculture and Environmental Sciences (CAES), University Mohammed VI Polytechnic (UM6P), 660 Lot, Ben Guerir, 43150 Morocco; 2 Center of Remote Sensing Applications (CRSA), College of Agriculture and Environmental Sciences (CAES), Mohammed VI Polytechnic University (UM6P), 660 Lot, Ben Guerir, 43150 Morocco; 3 Department of Earth Sciences, Faculty of Sciences and Techniques of Tangier (FST), Abdelmalek Essaadi University (UAE), Tangier, Morocco

**Keywords:** Land cover classification, Satellite Imagery, K-Means, NDVI, MNDWI, Scientific data, Computational science

## Abstract

This study presents a pixel-based unsupervised classification approach for mapping cultivated land using high-resolution imagery from the Moroccan Mohammed VI satellite. The proposed method integrates the K-means clustering algorithm with spectral features derived from vegetation indices, particularly the Normalized Difference Vegetation Index (NDVI) and the Modified Normalized Difference Water Index (MNDWI), together with the Near-Infrared (NIR) band. The output is a classified map composed of three classes: background, bare soil, and crop-dominated areas. The method was evaluated over a 175-hectare agricultural region in northern Morocco and achieved a relative error of 1.41%, significantly outperforming NIR threshold-based classification (7.2% error), NDVI-based classification (6.95%), and standard K-means classification using spectral bands only (5.47%). The results demonstrate the effectiveness of combining vegetation indices with unsupervised clustering and highlight the potential of the high-resolution satellite imagery for field-scale agricultural mapping, precision irrigation support, and sustainable land management.

## Introduction

Agriculture has always been a vital part of human life, playing a vital role in the maintenance of societies throughout history. With the continuous development of technology, agriculture has also evolved significantly. Innovations in various fields, such as embedded systems and robotics, have made farming operations more efficient. These technologies assist farmers with tasks such as planting, irrigation, and harvesting, ultimately improving productivity, reducing human labor, and promoting sustainability^1^.

One of the most significant advances in modern agriculture is the integration of artificial intelligence (AI) and computer science. These technologies are transforming agriculture by enabling farmers to make faster, better informed decisions. With the help of sensors and data collection tools, AI can analyze field data to improve crop yields, optimize resource management, and even predict weather conditions. These innovations help farmers adapt to climate change, improve food security, and adopt sustainable practices^2^.

In recent years, precision agriculture has emerged as a powerful approach in agriculture. Precision farming uses advanced technologies such as sensors, satellites, and drones to manage crops and fields with greater precision. Using data from these sources, farmers can monitor soil health, water use, and crop conditions, allowing for the application of treatments at optimal times, reducing waste (e.g., of water and fertilizers), and maximizing productivity^3^.

One of the key tools in precision farming is remote sensing from satellites and aircraft, as well as proximal sensing from drones (UAVs), which involve collecting data from the Earth’s surface using these platforms. Remote sensing captures spectral information that provides insights into crop health, soil conditions, water stress, and much more. It allows for the identification of healthy vegetation, the detection of water stress, and the early identification of pests and diseases that affect crops^4^.

Historically, remote sensing technologies were limited in resolution and data availability. However, with rapid advances in AI and satellite technology, there has been a significant increase in both the resolution and the quantity of available data. Modern satellites, such as those from the WorldView^5^ or Pleiades series^6^, now offer high-resolution images with a wide range of spectral data. While this high-resolution offer detailed imagery, data access is often restricted or costly, limiting widespread use in agricultural monitoring. This increased data availability has revolutionized the way we monitor agricultural fields. These advances allow for more accurate analysis and better decision-making in agricultural practices. In general, satellite imagery is a specialized field that involves several processes and steps in acquiring usable images. These images are captured through various sensors on satellites that collect data at different wavelengths. The data is pre-processed to ensure its accuracy, involving steps like calibration, geometric correction, and radiometric correction. These preprocessing steps ensure that the images are ready for detailed analysis^7^.

In reality, satellite images are typically multispectral, meaning that they consist of several bands beyond the traditional RGB (Red, Green, and Blue) bands. The additional spectral bands provide essential information for agricultural tasks such as assessing vegetation health, detecting water stress, soil analysis, and more. For example, vegetation indices like NDVI (Normalized Difference Vegetation Index) rely on these additional bands to assess the condition and growth of vegetation. An essential factor in satellite imagery is spatial resolution, which determines the level of detail visible in an image. Spatial resolution is defined by the size of each pixel in the image and plays a crucial role in the quality of the analysis. For example, free satellite systems like Sentinel and Landsat offer spatial resolutions greater than 10 meters, meaning each pixel represents a 10x10 meter area on the ground^8^. Although this resolution is useful for large-scale observations, it makes it challenging to detect smaller objects such as trees or small agricultural parcels^9^.

In contrast, commercial satellites such as Pleiades, WorldView, and GeoEye offer much higher spatial resolutions of less than 1 meter. These high-resolution images are particularly useful for detecting smaller objects, such as crops, small parcels, and even trees or plants. Using remote sensing data, several indices have been developed to extract valuable information about crop health, water stress, soil conditions, and more^10^. These indices are based on specific combinations of spectral bands, typically including the red, green, blue, and near-infrared bands, which are key to understanding the condition of vegetation and the environment (Table 1). These indices are invaluable for monitoring agricultural fields, improving crop health assessments, identifying areas affected by water stress, and improving resource management in precision farming. They also support various other applications, such as precision irrigation and weed detection, by helping differentiate between crops and unwanted plants^11^. On a larger scale, satellite-based indices can help governments monitor farmland over time, estimating crop yields, tracking food supply, and observing changes in land use. Indices such as the normalized difference water index (NDWI) are also useful for measuring soil moisture, detecting droughts, and managing water resources more effectively^13^.

**Table 1:** Common vegetation indices and their formulas.

Index name	Formula	Reference
NDVI (Normalized Difference Vegetation Index)	$\frac{(\text {NIR} - \text {Red})}{(\text {NIR} + \text {Red})}$	^14^
EVI (Enhanced Vegetation Index)	$\frac{(\text {NIR} - \text {Red})}{(\text {NIR} + C_1 \times \text {Red} - C_2 \times \text {Blue} + L)}$	^15^
SAVI (Soil Adjusted Vegetation Index)	$\frac{(\text {NIR} - \text {Red})}{(\text {NIR} + \text {Red} + L)} \times (1 + L)$	^16^
GCI (Green Chlorophyll Index)	$\frac{{NIR}}{{Green}} - 1$	^17^
CVI (Chlorophyll Vegetation Index)	$\frac{\text {NIR}}{(\text {Red} - \text {Green})}$	^18^
DVI (Difference Vegetation Index)	$\text {NIR} - \text {Red}$	^19^
NDWI (Normalized Difference Water Index)	$\frac{(\text {NIR} - \text {SWIR})}{(\text {NIR} + \text {SWIR})}$	^20^
MNDWI (Modified Normalized Difference Water Index)	$\frac{(\text {Green} - \text {NIR})}{(\text {Green} + \text {NIR})}$	^21^

**NIR**: Near-Infrared band.

**Red**: Red band, sensitive to chlorophyll absorption.

**Green**: Green band, sensitive to vegetation reflectance.

**Blue**: Blue band used in EVI computation.

**SWIR**: Shortwave Infrared band.

**C1, C2**: Empirical constants in EVI formulation.

**L**: Soil adjustment factor used in SAVI ($L = 0.5$,^16^). For EVI, $L = 1$, $C_1 = 6$, $C_2 = 7.5$^15^

These indices help distinguish different characteristics within farmland, making it easier to assess plant health, measure vegetation growth, and understand land use. They are particularly useful for automatically identifying various types of crops in satellite images^22^. This helps farmers efficiently allocate resources such as water and fertilizers, while also allowing for early detection of unhealthy or stressed crops to mitigate potential problems. In addition, satellite imagery and indices play an important role in precision irrigation, where drones or robots can target specific areas that need watering, thus conserving water in large farms^11^. They are also beneficial in weed detection, allowing farmers to identify and manage unwanted plants without using herbicides or damaging beneficial crops, etc.^12^

From the literature, the study of Giovos et al.^23^ presents an extensive review of VIs used in viticulture, highlighting different approaches and technologies. Sentinel-2 imagery was widely used due to its open-access spatial resolution, which allowed analysis of spatial variability within vineyards^26,27^. The authors observed that NDVI was the dominant index, appearing in 99 articles and is frequently used to delineate vineyard management zones and extract vine rows^28,29^. However, the resolution of Sentinel-2 poses a limitation, as the 10-meter pixel size does not eliminate the interference of the inner-row vegetation^30^. On the other hand, Ballesteros et al.^31^ integrated AI techniques, specifically artificial neural networks (ANN), to improve the prediction of vineyard yield using VIs from remote sensing imagery. Their approach improved prediction accuracy, achieving $R^{2}$ values between 0.6 and 0.9. This suggests that AI can significantly refine the interpretation of satellite-derived indices^32,33^.

Furthermore, Poblete et al.^34^ used multispectral UAV imagery in conjunction with ANN models to assess the state of vine water, obtaining an R2 of 0.93, which surpasses the performance of traditional regression methods^35^. A notable contribution from Segarra et al.^36^ involved integrating Sentinel-2 and Sentinel-1 synthetic aperture radar (SAR) data for soil moisture and irrigation management. This fusion approach extends beyond vegetation monitoring by incorporating soil parameters, providing a comprehensive decision support system^37^. In addition, hyperspectral remote sensing has been explored by Zarco-Tejada et al.^38^, who developed indices sensitive to chlorophyll concentration, outperforming traditional NDVI-based assessments to detect vine stress^39^. Regarding spectral bands, most studies used the NIR and red-edge bands due to their sensitivity to plant health and biomass^40,41^. The Green Normalized Difference Vegetation Index (GNDVI) and the Soil Adjusted Vegetation Index (SAVI) were frequently applied to compensate for soil background effects in medium-resolution images^42^. However, these papers indicate that while medium resolution satellites such as Sentinel-2 provide valuable information, high resolution imagery is superior for site-specific vineyard analysis^43^.

Despite their widespread use and effectiveness, vegetation indices such as NDVI, EVI, and SAVI have inherent limitations^44,45^. These indices are often crop-specific and may not perform equally well in different types of vegetation or agricultural systems^46^. For example, some crops with unique spectral signatures or growth patterns may not be accurately represented by existing indices. Furthermore, the spatial resolution of satellite imagery, even from high-resolution systems, can sometimes fail to capture fine-scale variations within fields, leading to incomplete or inaccurate assessments of crop health and land conditions^47^. Furthermore, environmental factors such as soil background effects, atmospheric conditions, and cloud cover can interfere with the accuracy of these indices, limiting their reliability in certain scenarios. Another challenge is the dynamic nature of agricultural landscapes, where changes in crop types, planting patterns, and land use can make existing indices less effective over time. These limitations highlight the need for more adaptive and robust methods to enhance the utility of vegetation indices in precision agriculture.

To address these challenges, this paper proposes combining unsupervised machine learning techniques, especially the K-Means clustering algorithm, with vegetation indices. The aim is to develop a pixel-based classification framework that integrates K-means clustering with spectral indices such as NDVI and MNDWI for improved vegetation and land cover analysis. The approach was tested using high-resolution imagery from the Moroccan Mohammed VI satellite to overcome the limitations of existing indices^48^ . The ultimate goal is to monitor and manage the land by distinguishing cultivated and uncultivated areas, identifying and classifying crops, and assessing the health of vegetation, including water stress and overall condition.

## Materials and methods

The data used in this study is a satellite image captured on 23 July 2020 by the Mohammed VI satellite system, a Moroccan Earth observation and reconnaissance satellite. The system consists of two satellites, Mohammed VI-A and Mohammed VI-B, designed by Thales Alenia Space (payload) and Airbus (satellite platform) for the Royal Center for Remote Sensing (CRTS) in Morocco. The first satellite, Mohammed VI-A, was launched on November 8, 2017, and the second, Mohammed VI-B, on November 21, 2018, both aboard a Vega launcher from the Kourou Space Center^47^.

These satellites operate in a sun-synchronous orbit at an altitude of 647 km, with an inclination of 98.0$^\circ$, ensuring global coverage and a revisit time of less than 24 hours for strategic areas. Each satellite is based on the AstroSat-1000 platform, ensuring reliable operation for Earth observation applications. The system has a 5-year design lifespan and provides very high-resolution imagery, capturing data in four spectral bands: Red, Green, Blue (RGB) and Near-Infrared (NIR), with a native pan-sharpened spatial resolution of 0.5 meters^49^. This high-resolution capability supports applications ranging from parcel-level agricultural monitoring to regional-scale land use assessment.

The study region is located in northern Morocco, specifically in the Fes-Meknes region, with coordinates 34.424997$^\circ$N latitude and -5.661996$^\circ$W longitude. It covers a total area of 175 hectares, comprising a mix of uncultivated land, sparsely vegetated areas, and parcels dedicated to a variety of seasonal and permanent crops, including cereals, maize, olives, almonds, grapes, carob, potatoes, onions, and garlic^50^. The region was selected for its diversity of land covers and crop types, as well as its suitability for regional-scale agricultural monitoring, which refers to the assessment of cultivated areas across hundreds to thousands of hectares for purposes such as crop inventory, irrigation planning, and sustainable land management.

**Figure 1:**
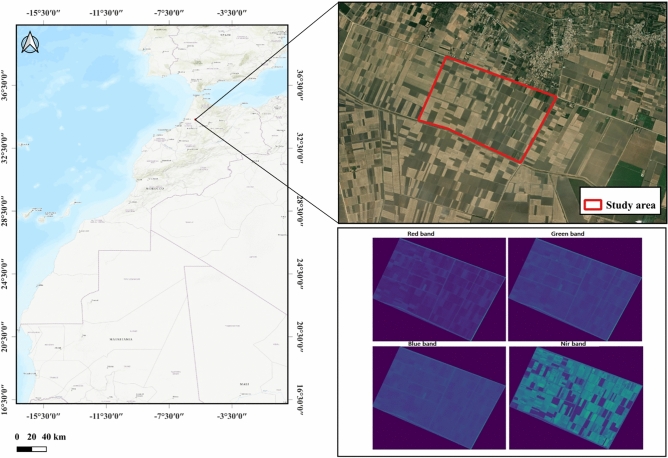
Study region.

Figure 1 shows our study region along with the image used, which consists of four bands: RGB and the NIR band. The image captures both cultivated and uncultivated parcels, with the NIR band expected to primarily enhance the visibility of cultivated areas.

### K-means algorithm

K-means is one of the most widely used algorithms in unsupervised machine learning^51^. It is particularly effective in clustering large datasets by partitioning data points into *K* clusters based on similarity. The algorithm follows an iterative approach to refine cluster assignments and optimize cluster centroids.

The K-means algorithm proceeds as follows: 1. Choose the number of clusters, *K*. 2. Initialize centroids *K* in random clusters. 3. Assign each data point to the nearest cluster centroid based on the Euclidean distance (Equation 1). 4. Compute new centroids by averaging the data points within each cluster. 5. Repeat steps 3 and 4 until the centroids no longer change or the maximum number of iterations is reached.

The Euclidean distance between a data point $x_i = (x_{i1}, x_{i2},..., x_{id})$ and a cluster centroid $c_j = (c_{j1}, c_{j2},..., c_{jd})$ in a *d*-dimensional space is given by:

1 $$\begin{aligned} d(x_i, c_j) = \sqrt{\sum _{k=1}^{d} (x_{ik} - c_{jk})^2} \end{aligned}$$

The algorithm stops when the centroids stabilize, meaning they no longer shift significantly between iterations. The K-means algorithm is illustrated in Algorithm 1.

**Algorithm 1:**
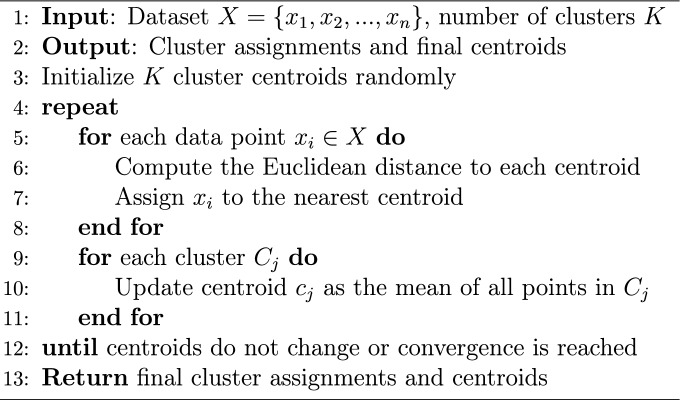
K-means Clustering Algorithm

In the field of image processing, K-means is used to segment an image into multiple groups of pixels based on their Euclidean distance. This means that pixels with similar characteristics and located close to each other are grouped into the same cluster.

In agriculture, using spectral images, K-means can be an effective algorithm to detect and classify crops. It helps distinguish different types of vegetation, separate cultivated areas from uncultivated land, and identify specific crop regions based on their spectral information.

### Ground truth data preparation

**Figure 2:**
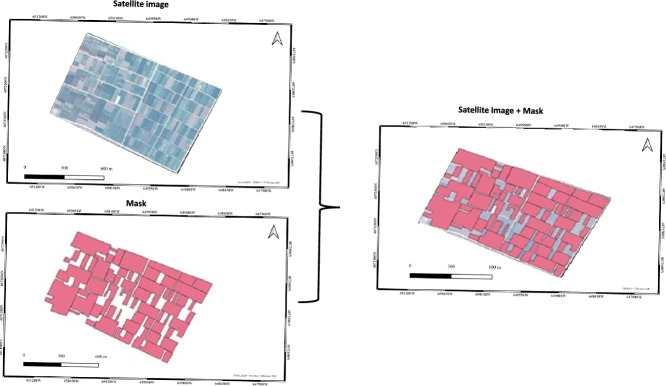
Data labeling process-integration of a pixel-based mask with a satellite image.

In order to evaluate the performance of our porposed method, we manually delineated polygons that correspond to cultivated parcels using open-source QGIS software (Quantum Geographic Information System). This process involves loading the satellite image into QGIS by creating a new project and adding the raster layer that contains the cultivated parcels. Once the image is displayed, a new vector layer is created to store the manually traced polygons. The geometry type is set to polygon and an appropriate coordinate reference system (CRS) is selected that matches the satellite image. Using the digitization tools in QGIS, the cultivated areas are carefully outlined to ensure an accurate representation of the ground truth. Each polygon is labeled and stored within the vector layer, which will serve as a reference for evaluating the precision of the proposed method (Fig. 2).

While independent field surveys or cadastral data would provide additional validation strength, expert manual digitization from the same high-resolution image offers a precise and consistent reference layer suitable for a controlled comparison of pixel-based classification methods, all of which are derived from the same spectral source.

The resulting data set is then used to compare the classification results, allowing an assessment of how well the proposed approach identifies the cultivated parcels.

### Implementation and computational environment

The proposed methodology was implemented in a Google Colab environment using Python 3.10. The following Python libraries were used for data processing and analysis: rasterio and GDAL for reading and manipulating geospatial raster data, NumPy for numerical array operations, and scikit-learn for machine learning utilities.

All computations were performed using the T4 GPU runtime available in Google Colab, with 16 GB of RAM^52^.

## Approach

Our approach focuses on developing a spectral clustering framework that integrates NDVI, MNDWI, and the NIR band with the K-means algorithm. Since the Mohammed VI satellite lacks the SWIR bands required for the traditional NDWI, we employ MNDWI which operates on Green and NIR bands as a spectral contrast index to enhance discrimination between vegetated and non-vegetated areas based on moisture-related reflectance differences. While the NIR band is valuable for detecting vegetation presence, its utility for precise separation between soil and crops is limited without complementary spectral information such as red-edge or SWIR bands. Therefore, our objective is to leverage the complementary information from NDVI (sensitive to chlorophyll content) and MNDWI (sensitive to vegetation water status) alongside the NIR reflectance, and to apply unsupervised K-means clustering to achieve a more robust and accurate pixel-based classification than is possible with simple NIR thresholding or single-index methods.

**Figure 3:**
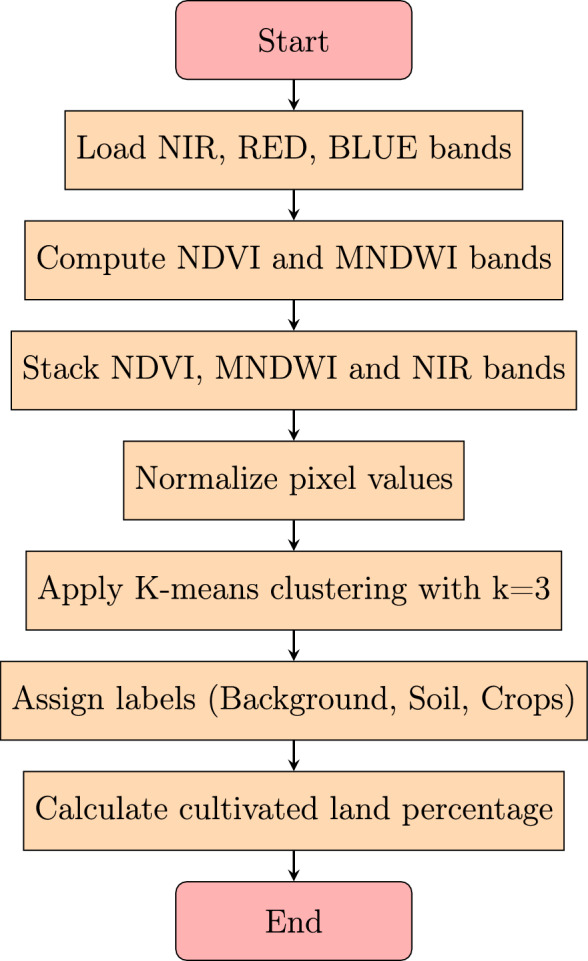
Workflow of the proposed approach integrating NDVI, NIR, and K-means clustering.

The workflow of the approach is illustrated in Fig. 3.

The proposed approach consists of the following steps:

- **Preprocessing:** The input satellite image is loaded, and the necessary spectral bands are extracted. Specifically, the NIR band, the RED band, and the BLUE band are retrieved. The data is converted to floating-point format for numerical processing.
- **Computation of Vegetation Indices:** Using the extracted bands, the NDVI and MNDWI indices are computed.
- **Feature Stacking and Normalization:** The computed NDVI and MNDWI values, along with the original NIR band, are stacked together to form a three-band spectral image. The pixel values are then normalized between 0 and 1 to ensure uniform feature scaling.
- **K-means Clustering for Land Classification:** The K-means algorithm is applied to segment the image into three classes:
  - **Background** (background of the image)
  - **Soil** (bare land or uncultivated regions)
  - **Crops** (vegetated and cultivated areas)

The number of clusters was fixed to $k=3$ to correspond to the three land-cover classes required for agricultural monitoring: Background, Soil, and Crops. This predefined value ensures that the clustering results remain semantically consistent with the study objectives and avoids the generation of ambiguous or redundant clusters that may arise with larger values of *k*.

## Results and discussion

The performance of the proposed approach was evaluated against the manually digitized ground truth data described in Section 2.2. The cultivated area percentage was calculated by overlaying the ground truth mask on the satellite image to isolate cultivated pixels, summing their values to obtain the total cultivated area, and deriving the total image area from the spatial resolution and pixel count. Based on this calculation, the cultivated area was found to constitute 71.07% of the total study region, as determined by the following formula: 2.

2 $$\begin{aligned} \text {Percentage of cultivated area} = \left( \frac{\text {Cultivated Land Area}}{\text {Total Land Area}} \right) \times 100 \end{aligned}$$

Our objective is to compare different pixel-based classification approaches, including NIR-based pixel classification, NDVI-based pixel classification, K-means clustering, and our proposed spectral K-means method, to evaluate their effectiveness in accurately identifying cultivated areas.

### NIR thresholding pixel-based classification

The first approach tested is based on a simple thresholding of the Near-Infrared (NIR) band. The pixel-based classification process classifies each pixel into one of three categories: background, soil, or crops. The classification is performed using predefined threshold values, where pixels with an intensity of zero are considered background, those with values between 0 and 700 correspond to the soil, and pixels with values greater than 700 are classified as crops^53–55^. The classification function assigns each pixel to its respective category, producing a segmented image that highlights cultivated areas.

**Figure 4:**
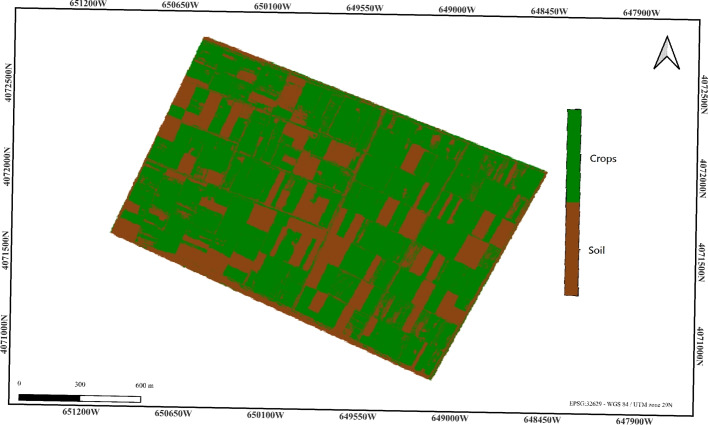
NIR thresholding classification result (cultivated land: 65.59%). Color key: White = Background, Brown = Soil, Green = Crops.

As a result, this method estimates the percentage of cultivated land as 65.59%, which is significantly lower than the reference value of 71.07%. This suggests that the NIR thresholding approach underestimates the cultivated land area, likely due to its inability to account for variations in vegetation reflectance. This limitation demonstrates the need for more advanced pixel-based classification techniques to improve precision (Fig. 4).

### NDVI-based pixel classification

In the second approach, the Normalized Difference Vegetation Index (NDVI) is used to segment the image into three categories: background, soil, and crops (Table 1).

The pixel-based classification process classifies pixels based on the NDVI value, where values close to zero correspond to the background, values between -1 and 0.2 correspond to the soil, and values greater than 0.2 are classified as crops^56,57^. This classification provides a more nuanced differentiation between land types compared to the simple NIR thresholding method. The NDVI method estimates the percentage of cultivated land at 66.13%, which is an acceptable result, but it is not entirely satisfactory compared to the reference value of 71.07%. This suggests that while the NDVI approach improves on the NIR thresholding method, there is still room for improvement in the accuracy of land use classification.

**Figure 5:**
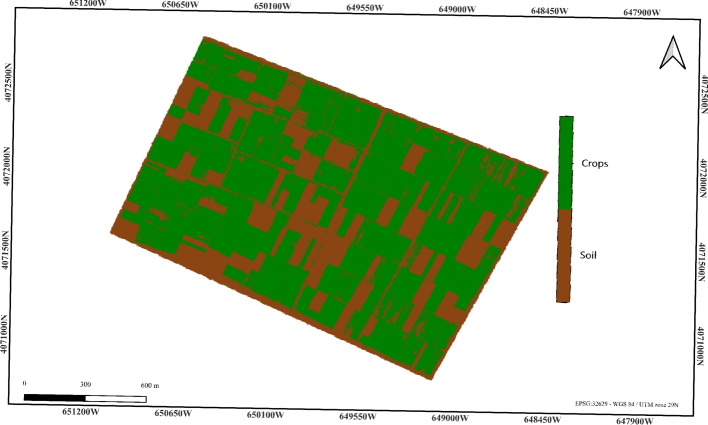
NDVI-based classification result (cultivated land: 66.13%). Color key: White = Background, Brown = Soil, Green = Crops.

Figure 5 displays the NDVI-based classification, highlighting the distribution of cultivated land, soil, and background.

### K-means pixel-based classification

**Figure 6:**
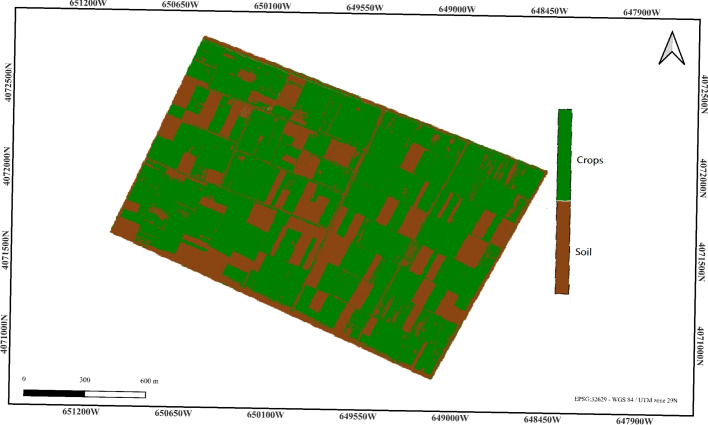
K-means classification result (cultivated land: 67.18%). Color key: White = Background, Brown = Soil, Green = Crops.

The third approach applied to segment the image is the K-Means clustering, which divides the data into three distinct classes: background, soil, and crops. The K-Means algorithm was configured with three groups representing these land categories. The spectral image was derived by stacking the Near-Infrared (NIR), Red, and Blue bands, which were normalized to a range of [0, 1] to ensure consistent scaling of the features before clustering. This normalization was chosen to preserve the original distribution of spectral values while scaling them to a common range, which is suitable for K-means clustering applied to optical remote sensing data. The number of groups, k = 3, was chosen based on the assumption of three main land categories: background, soil, and crops. The percentage of cultivated land estimated using K-Means is 67.18%, which shows a reasonable estimate but still falls short compared to the reference value of 71.07%. This discrepancy suggests that while the K-Means pixel-based classification provides a more accurate than the previous methods, there are still challenges in perfectly distinguishing the cultivated land, possibly due to variations in reflectance or overlap in spectral features between soil and crops. Despite this, the result is closer to the reference compared to other approaches, indicating that K-Means pixel-based classification is a promising method for land classification in this case. Figure 6 shows the pixel-based classification output.

### Spectral K-means pixel-based classification

In this approach, we combine three key spectral indices: NDVI, NIR, and MNDWI with K-Means clustering to improve the precision of land classification. The Near-Infrared (NIR) band and the Red and Blue bands are used to compute two vegetation indices: the NDVI and MNDWI (Table 1.

While MNDWI was originally designed for water body detection, its spectral contrast between Green and NIR bands provides complementary information that helps separate vegetated pixels from bare soil and background in our classification framework. It is used here as a spectral discriminator rather than a direct measure of vegetation water content.

The three bands are stacked to create a spectral image, which is then reshaped into a 2D array for clustering. To improve the clustering process, the pixel values are normalized in the range [0, 1], ensuring that each feature contributes equally to the clustering algorithm. And the K-Means clustering is applied with k=3, where the three clusters correspond to the background, soil, and crops.

**Figure 7:**
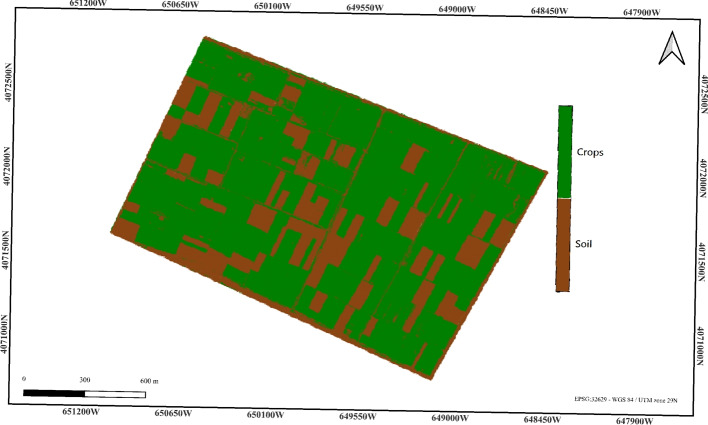
Proposed spectral K-means classification result (cultivated land: 72.07%). Color key: White = Background, Brown = Soil, Green = Crops.

The results indicate that the percentage of cultivated land is 72.07%, which is a promising result, as it is relatively close to the reference value, with only a small deviation (Fig. 7).

To compare the errors of the three methods, equation 3 presents the formula used to calculate the error between the detected area on the obtained map and the actual area of the ground truth.

3 $$\begin{aligned} \text {Error} = \left| \frac{\text {Detected Area} - \text {Actual Area}}{\text {Actual Area}} \right| \times 100 \end{aligned}$$

This relative error reflects the accuracy of cultivated area estimation and is directly related to overall classification performance. Lower error values indicate better alignment with the ground truth reference data.

The comparison results are summarized in Table 2, which illustrates the performance of different methods in the detection of cultivated land. It is evident that the spectral K-Means method performs significantly better than the traditional NIR threshold and NDVI pixel classification techniques. This suggests that combining spectral indices (NDVI and MNDWI) with K-Means clustering provides a robust solution for land classification and vegetation monitoring.

It is important to note that our validation is based on reference data derived from the same image used for classification. This approach ensures a fair and consistent benchmark for comparing spectral-based methods but highlights the need for future validation with independent datasets, such as field surveys or multi-temporal imagery, to assess operational robustness across different regions and seasons.

**Table 2:** Classification error comparison of different pixel-based methods for cultivated land detection.

Method	Error (%)
NIR Threshold Method	7.2
NDVI pixel classification	6.95
K-Means pixel classification	5.47
Proposed spectral K-Means	1.41

Many studies have focused on developing pixel-based classification and mapping for cultivated regions using satellite imagery. Classical methods, such as machine learning and object-based image analysis (OBIA), have been widely used because of their simplicity and computational efficiency. For example, Yan and Roy^58^ applied a phenology-based classification using multitemporal Landsat data to estimate crop field sizes in the United States, achieving general precision 85%. Similarly, Ouzemou et al.^59^ used Landsat-8 OLI NDVI time series data to detect crops in an irrigated region in Morocco using Support Vector Machine (SVM) and the Spectral Angle Mapper (SAM). Their results showed a 86% accuracy based on SVM, while SAM performed significantly worse at 57%. However, these approaches are better suited for large parcels because of the low resolution of satellite images and the reliance on basic techniques.

Other studies have explored OBIA-based approaches, such as Watkins and van Niekerk^60^, who compared different OBIA methods for field boundary delineation using multitemporal Sentinel-2 imagery. Their findings showed that OBIA provides better pixel classification than pixel-based methods but requires careful parameter tuning, which can limit its scalability. Although classical methods are computationally efficient and do not require large labeled datasets, they often suffer from manual parameter adjustments and lower classification accuracy, making them less robust for large-scale applications.

In contrast, deep learning approaches have demonstrated good performance in agricultural pixel-based classification in the last few years. Waldner and Diakogiannis^61^ developed DECODE, a deep learning-based model using FracTAL ResUNet, to extract field boundaries from Sentinel-2 images. Their model achieved 87% pixel-based accuracy and 88% field-based accuracy, significantly outperforming traditional pixel-based classification techniques. Similarly, Masoud et al.^62^ proposed a Fully Convolutional Network (FCN) with a super-resolution contour detector, which improved field boundary detection using Sentinel-2 images. Another deep learning study by Persello et al.^63^ applied an encoder-decoder FCN combined with combinatorial grouping to delineate agricultural fields in smallholder farms in Nigeria and Mali using WorldView-2 images, achieving F-scores above 70%, surpassing traditional contour detection methods.

As summarized in Table 3, most approaches rely on low-resolution free satellite imagery, which can limit their accuracy. Supervised machine learning and deep learning models are the most commonly used, as they have demonstrated strong results in recent years. However, deep learning techniques require large labeled datasets and significant computational resources, making them difficult to apply in regions where training data is scarce.

To address these challenges, our approach uses high-resolution satellite imagery (0.5 meters), which, while not free, remains cost-effective. Unlike traditional supervised machine learning or deep learning methods, our approach does not require labeled data. Instead, it applies unsupervised techniques to extract valuable spectral information directly from the satellite images. Among the tested methods, the K-means clustering of combined spectral indices (NDVI, MNDWI, and NIR) provided a very good score that closely matches the reference data. This applicative integration of standard clustering with established spectral features offers a practical and robust solution for land classification. It presents significant benefits for farmers and decision-makers, particularly in large-scale agricultural management. The ability to accurately distinguish cultivated and uncultivated areas, monitor vegetation health, and track crop yields improves precision agriculture by offering timely information on crop conditions.

Ultimately, our approach highlights the potential of unsupervised spectral clustering in agricultural mapping. By eliminating the need for labeled training data, it reduces manual effort and computational costs, making it an accessible and efficient solution for large-scale agricultural monitoring. This method not only improves crop management, but also supports sustainable farming practices by enabling better resource allocation and land use planning.

**Table 3:** Land cultivated mapping results.

Method	Satellite	Result	Advantages	Limitations
SVM^59^	Landsat-8	85.27% Accuracy	Simple, no large dataset needed	Manual feature selection, low pixel-based classification accuracy
Phenology-based^58^	Landsat Multi-Temp.	85% Accuracy	Large-scale mapping	Sensitive to seasonal changes, boundary imprecision
OBIA^60^	Sentinel-2	-	Uses spatial and spectral data	Requires parameter tuning
FracTAL ResUNet^61^	Sentinel-2	88% Accuracy	High accuracy, robust pixel-based classification	Needs large labeled data
FCN + Super-Res.^62^	Sentinel-2	-	Precise boundaries	Limited by Sentinel-2 resolution
Encoder-Decoder FCN^63^	WorldView-2/3	70% F-score	Works well on irregular fields	Computationally expensive
**Spectral K-means (Our approach)**	Mohammed VI	1.41% Error	Accurate for small/large areas, cost-effective, no labels needed	Limited public/open-access availability; data accessible via CRTS under institutional agreement.

## Conclusion

In this study, we proposed a new approach for monitoring cultivated land and vegetation in a large area using high-resolution satellite imagery from the Mohammed VI satellite system. By integrating spectral indices (NDVI and MNDWI) and the idea behind the K-means clustering algorithm, we achieved an estimated percentage of cultivated land with an error of 1.41%. This score demonstrates the reliability of our method for large-scale agricultural monitoring.

The benefits of this method are particularly valuable for farmers and decision makers managing large agricultural areas. By distinguishing between cultivated and non-cultivated parcels, our approach allows for better monitoring of vegetation health, crop yields, and land use efficiency. This supports precision agriculture by helping farmers allocate resources more effectively, improve crop management, and increase productivity. Furthermore, as high-resolution imagery from the Mohammed VI system is made available through the CRTS company in Morocco under a formal pricing structure, the proposed method offers a practical and powerful tool for large-scale agricultural monitoring and decision-making in Morocco.

Looking ahead, our goal is to extend this work to more specific applications, such as gaining detailed information on individual trees, enabling early detection of stress conditions, and facilitating targeted interventions to improve crop health and productivity. Integrating unsupervised machine learning with high-resolution spectral imagery presents a promising direction for advancing precision agriculture and supporting sustainable land management practices.

## Data Availability

The datasets generated or/and analysed during the current study are not publicly available due to restrictions by the Royal Center for Remote Sensing(CRTS) of Morocco, but are available from the corresponding author upon reasonable request.
